# Integrative transcriptome analysis reveals alternative polyadenylation potentially contributes to GCRV early infection

**DOI:** 10.3389/fmicb.2023.1269164

**Published:** 2023-11-03

**Authors:** Sheng Tan, Jie Zhang, Yonglin Peng, Wenfei Du, Jingxuan Yan, Qin Fang

**Affiliations:** ^1^Key Laboratory of Systems Biomedicine (Ministry of Education), Shanghai, Center for Systems Biomedicine, Shanghai Jiao Tong University, Shanghai, China; ^2^State Key Laboratory of Virology, Wuhan Institute of Virology, Chinese Academy of Sciences, Wuhan, China; ^3^Institute of Hydrobiology, Chinese Academy of Sciences, Wuhan, China; ^4^Bio-ID Center, School of Biomedical Engineering, Shanghai Jiao Tong University, Shanghai, China

**Keywords:** grass carp reovirus, Aquareovirus genus, *Ctenopharyngodon idellus*, DNA methylation, alternative polyadenylation

## Abstract

**Introduction:**

Grass carp reovirus (GCRV), a member of the *Aquareovirus genus* in the *Reoviridae* family, is considered to be the most pathogenic aquareovirus. Productive viral infection requires extensive interactions between viruses and host cells. However, the molecular mechanisms underlying GCRV early infection remains elusive.

**Methods:**

In this study we performed transcriptome and DNA methylome analyses with *Ctenopharyngodon idellus* kidney (CIK) cells infected with GCRV at 0, 4, and 8 h post infection (hpi), respectively.

**Results:**

We found that at early infection stage the differentially expressed genes related to defense response and immune response in CIK cells are activated. Although DNA methylation pattern of CIK cells 8 hpi is similar to mock-infected cells, we identified a considerable number of genes that selectively utilize alternative polyadenylation sites. Particularly, we found that biological processes of cytoskeleton organization and regulation of microtubule polymerization are statistically enriched in the genes with altered 3’UTRs.

**Discussion:**

Our results suggest that alternative polyadenylation potentially contributes to GCRV early infection.

## Introduction

Aquareoviruses are nonenveloped viruses and classified within the family Reoviridae, a family of double-stranded RNA virus composed of aquareoviruses, mammalian reoviruses (MRV), and the other 13 genera. Aquareoviruses cause infection in aquatic organisms including bony fish, shellfish, and crustacean worldwide (Lupianni et al., 1995). Although most aquareoviruses are isolated from seemingly healthy fish and do not give rise to high mortalities, grass carp reovirus (GCRV) is recognized to be most pathogenic among the isolated aquareoviruses (Rangel et al., 1999). GCRV can cause serious hemorrhagic disease in aquatic organisms. Our previous studies have shown that GCRV can induce cell–cell fusion and produce characteristic cytopathic effect (CPE) consisting of large syncytia within infected cultures (Fang et al., 1989; Ke et al., 1990), and it has been extensively used to understand aquareovirus molecular and structural biology. Seven structural (VP1–VP7) and six nonstructural proteins (NS12, NS16, NS26, NS31, NS38, and NS80) of GCRV have been well identified (Guo et al., 2013; Yan et al., 2015). Comparative proteomic analysis of lysine acetylation in fish Ctenopharyngodon idellus kidney (CIK) cells reveals the proteome-wide changes in host cell acetylome with GCRV infection (Guo et al., 2017). MRV can cause chronic infection. It has been revealed that there is a close molecular evolutionary relationship between aquareoviruses and mammalian orthoreoviruses. In addition to morphological similarity, GCRV and MRV share high amino acid conservation. A better knowledge of the interaction during early infection stage between GCRV and host cells will help the understanding molecular pathogenesis of the aquareovirus and other members in the family Reoviridae.

Accumulating evidence has demonstrated that epigenetics is actively involved in host-virus interaction. Epigenetic trait is defined as a stably heritable phenotype resulting from changes in a chromosome without alterations in the DNA sequence (Berger et al., 2009). These chromosomal changes include methylation of cytosine in CpG dinucleotides (often referred to as DNA methylation) and other posttranslational covalent modifications to histones, such as methylation, acetylation, and ubiquitylation. The epigenetic modifications are associated with structural organization of chromatin and transcriptional activities of the affected genes. As intracellular parasites, viruses develop various ways of remodeling epigenetic alterations to facilitate their infection and replication. Through inducing DNA methylation changes in host cells viruses epigenetically manipulate host functions upon virus infection. For instance, Epstein–Barr virus (EBV) infection activates cellular DNA methyltransferases and results in aberrant DNA methylation in host cells (Tsai et al., 2006; Hino et al., 2009). HIV infection can also trigger the differential DNA methylation at cis-regulatory regions of host genomic DNA and inhibit the function of T cells (Pion et al., 2013; Youngblood et al., 2013). Nevertheless, the influence on cellular DNA methylation during GCRV infection remains to be further characterized.

In addition to epigenetic modifications, formation of stress granules is also actively involved in the interaction between viruses and host cells. It has been recognized that the innate immune response of host cells is triggered by upon virus infection to prevent pathogen invasion, partially through stress granules. Some components of stress granules have been identified, such as T-cell-restricted intracellular antigen 1 (TIA-1), TIA-1-related protein (TIAR), Ras GTPase-activating protein-binding proteins (G3BPs)and poly(A)-binding proteins (PABPs). PABPs are a family of RNA recognition motif (RRM)-containing proteins that bind poly(A) tail and regulate translation and stability of mRNA. The previous report has demonstrated that alternative polyadenylation (APA) plays an important role in the antiviral innate immune response (Jia et al., 2017). However, it remains unclear whether APA of host cells is involved in GCRV infection. Thus, in this study we carried out integrative analyses of transcriptome, DNA methylome and APA in GCRV-infected CIK cells for understanding the molecular events in GCRV early infection.

## Materials and methods

### Cells, virus and infection assays

CIK cells, purchased from the China Center for Type Culture Collection (CCTCC, 4201FIS-CCTCC00086), were grown in minimum essential medium (MEM; Gibco-BRL) supplemented with 10% fetal bovine serum (FBS), 100 mg/mL penicillin, and 100 mg/mL streptomycin at 28°C. Grass carp reovirus (strain GCRV-873), previously isolated and stored in the author’s laboratory, was propagated in CIK cells with Eagle’s MEM supplemented with 2% FBS (MEM-2) as previously described (Fang et al., 1989).

### Viral infection, cytopathic effect observation and plaque assay

The infection assays were carried out as we described previously (Zhang et al., 2019). Briefly, the 80% confluent CIK cells in T-25 flask (Corning Inc., Corning, NY, United States) with a concentration of 2 × 10^6^ cells/ml were inoculated with GCRV at a multiplicity of infection (MOI) of 1 in serum-free MEM medium at 28°C for 1 h following the method as previously reported (Guo et al., 2017). For comparison, the mock-infected cells were treated with same amount of medium in the same conditions. Upon adsorption, cells were washed with phosphate-buffered saline (PBS) to remove non-adsorbed virions. The infected cells were maintained in MEM-2 at 28°C and harvested at 0 (mock), 4 and 8 h post infection, respectively. When initial cytopathic effects were observed, the infected cells and mock-infected cells were prepared and harvested for further transcriptome analyses. Three rounds of independent experiments were performed. For MOI determination, plaque assays were done according to our previously described method (Yan et al., 2015; Zhang et al., 2018).

### RNA isolation, RNA-seq library construction and deep sequencing

CIK Cells were infected by GCRV for 0, 4 and 8 h, respectively. Total RNA was extracted with Trizol reagent (Invitrogen, United States), which was further treated with RNase-free DNase to remove genomic DNA. mRNA was purified with poly(dT) oligo-attached magnetic beads and broken down into 200 ~ 400 bp fragments. A strand-specific RNA-seq library was constructed with NEBNext Ultra Directional RNA Library Prep Kit (NEB, New England, United States). Briefly, the fragmented mRNA was reversely transcribed into cDNA with random primers and then the second-strand cDNA was generated. The resulting double-strand DNA fragments were purified with AMPure beads (Beckman Coulter, Brea, CA, United States) and ligated with Illumina adapters. The ligation products were purified by agarose gel electrophoresis to remove adapter dimmers, which were subsequently subjected to HiSeq X sequencing (Illumina, San Diego, CA, United States). The raw sequencing data could be obtained in the EMBL database^1^ under accession number E-MTAB-13002.^2^

### MeDIP-seq library construction and deep sequencing

Genomic DNA of CIK cells was extracted using GenElute^™^ Mammalian Genomic DNA Miniprep Kit (Sigma, United States). DNA was randomly sheared into fragments of 200 ~ 500 bp and used for library preparation with NEBNext^®^ Ultra^™^ II DNA Library Prep Kit for Illumina (NEB), the resulting libraries were purified with 1 × Agencourt AMPure XP beads (Beckman Coulter). The immunoprecipitation procedure was basically performed according to a previous MeDIP protocol (Taiwo et al., 2012). Briefly, the library DNA was denatured at 95°C for 10 min and immediately placed into an ice for 10 min, 1/10 volume of denatured product was set aside as Input. The Protein A + G magnetic beads (Millipore, United States) were incubated with 5-Methylcytosine (5-mC) monoclonal antibody (Epigentek) at 4°C for 2 h and the library was incubated with antibody-bead complexes at 4°C overnight with a slight rotation. The dynabead-antibody-methylated DNA complexes were washed three times, followed by proteinase K (Thermo scientific) treatment for 3 h at 55°C. The immunoprecipitated DNA was extracted by phenol/chloroform/isoamylalcohol, followed by ethanol precipitation, and resuspended in EB buffer (10 mM Tris–HCl pH 8.0). The enriched methylated DNA and Input DNA were amplified using Q5 High-Fidelity DNA Polymerase (NEB), and subject to Illumina sequencing platforms. The raw sequencing data could be obtained in the EMBL database (see Footnote 1) under accession number E-MTAB-13003.^4^

### Bioinformatics analysis

The raw reads with low quality and the adapter sequences of RNA-seq and MeDIP-seq data were removed using Cutadapt v4.1 (Kechin et al., 2017). For RNA-seq data, clean reads were mapped to the grass carp reference genome (Wang et al., 2015) using Hisat v2.2.1 (Kim et al., 2019). The Subread toolkits was used to quantify read counts for genes (Liao et al., 2014), and reads per kilobase of transcript per million mapped reads (RPKM) were calculated as expression levels. Differential expression analysis was performed using the edgeR package in R platform v3.6.3 (Robinson et al., 2010). Those genes with an value of *p* < 0.05 and fold change >1.5 were regarded as differentially expressed genes (DEGs). For MeDIP-seq data, clean reads were mapped to the grass carp reference genome using Bowtie v2.4.5 (Langmead and Salzberg, 2012). PCR duplicate reads were removed with Picard v2.27.4.^6^ DNA methylation peaks were called with MACS2 with deduplicated alignments (Zhang et al., 2008) and the differentially methylated regions (DMRs) were identified with DiffBind and DESeq2 packages (Anders and Huber, 2010). Functional enrichment analysis was performed with DAVID.^7^

### Analysis of APA with RNA-seq data

The APAs were identified by using DaPars algorithm (Masamha et al., 2014) based on RNA-seq data. Briefly, the observed sequence coverage was represented as a linear combination of annotated 3’UTRs. For each transcript with annotated proximal adenylation site (PAS), a regression model was used to infer the end point of alternative novel PAS within this 3’ UTR at single nucleotide resolution, by minimizing the deviation between the observed read coverage and the expected read coverage based on a two-PAS model in both mock-infected and GCRV-infected samples simultaneously. A percentage dPAS usage index (PDUI) was utilized to define shortening (negative index) or lengthening (positive index) of 3’UTR and thus capable of quantifying the degree of difference in 3’UTR usage between mock-infected and GCRV-infected CIK cells. The greater PDUI means that the more distal PAS of a given transcript is used and vice versa.

## Results

### Grass carp reovirus infection-induced cytopathic effects at early stage

**Figure 1:**
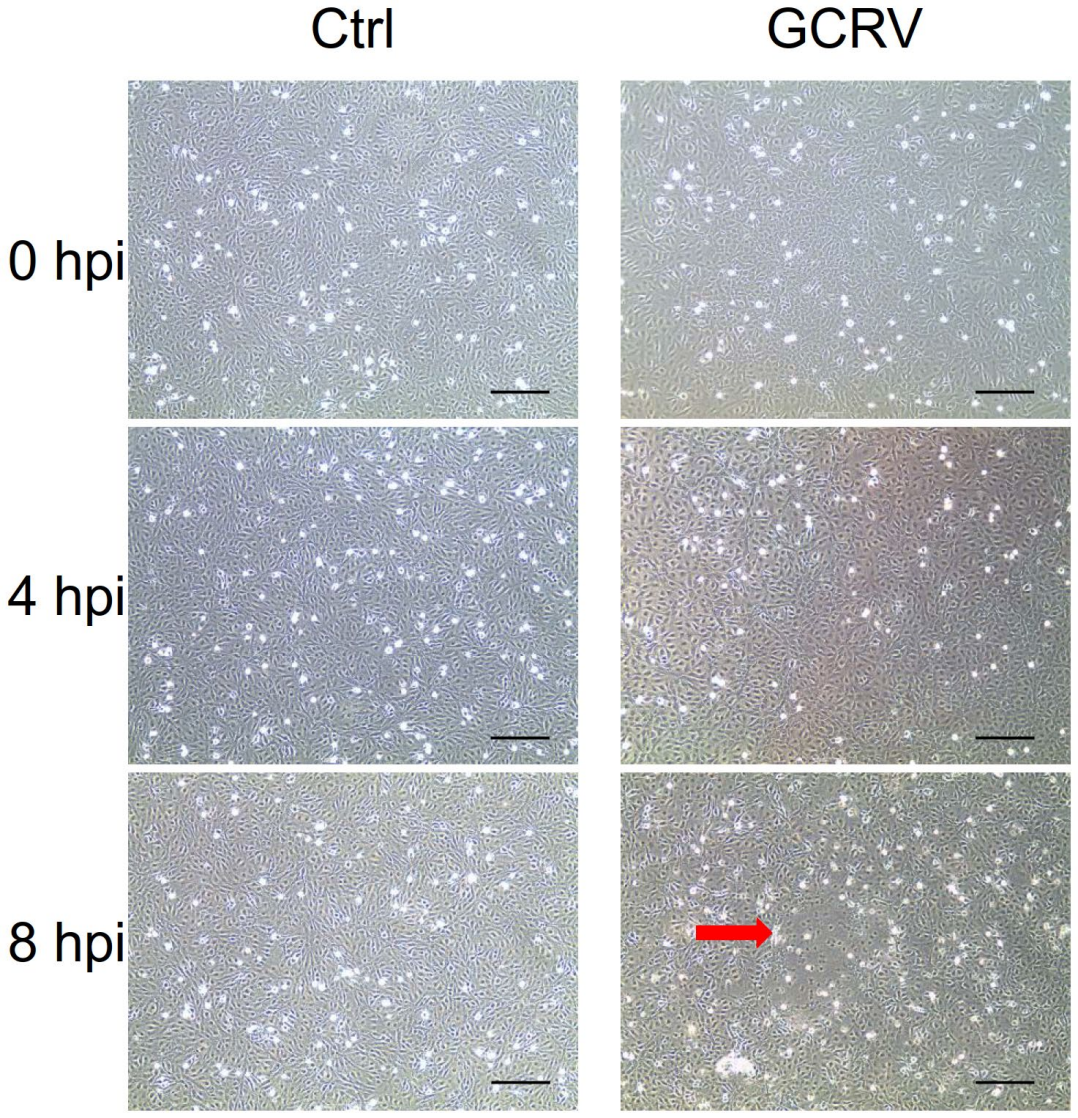
Grass carp reovirus (GCRV)-induced cytopathic effect in CIK cells at different time points. CIK cells were mock-infected (Left panel) or infected with GCRV(Right panel), and the images were taken at 0, 4 and 8 hpi, respectively. Red arrow shows the representative CPE. Sale bar: 200 μm.

To characterize the interaction of GCRV and host cells for integrative analyses of transcriptome in GCRV-infected CIK cells, we firstly carefully examined the cytopathic effects induced by GCRV infection at early stage. In both mock-infected cells and GCRV-infected cells at 4 h, we did not observe obvious CPE. As infection progressed, we detected an initial characteristic CPE on the monolayers of CIK cells at 8 h post infection (hpi) by comparing to mock-infected cell (Figure 1), which suggests that efficient infection was obtained, and the harvested infected cell lysates were suitable for follow-up transcriptome related assays.

### Transcription program associated with grass carp reovirus early infection

To detect the molecular events at the GCRV early infection, we performed RNA-seq analysis of CIK cells 0, 4 h and 8 hpi. Totally we generated 197.5 millions raw sequencing reads in the groups of mock, 4 and 8 hpi. Among these reads 94.7% are mappable and are used for downstream analysis.

**Figure 2:**
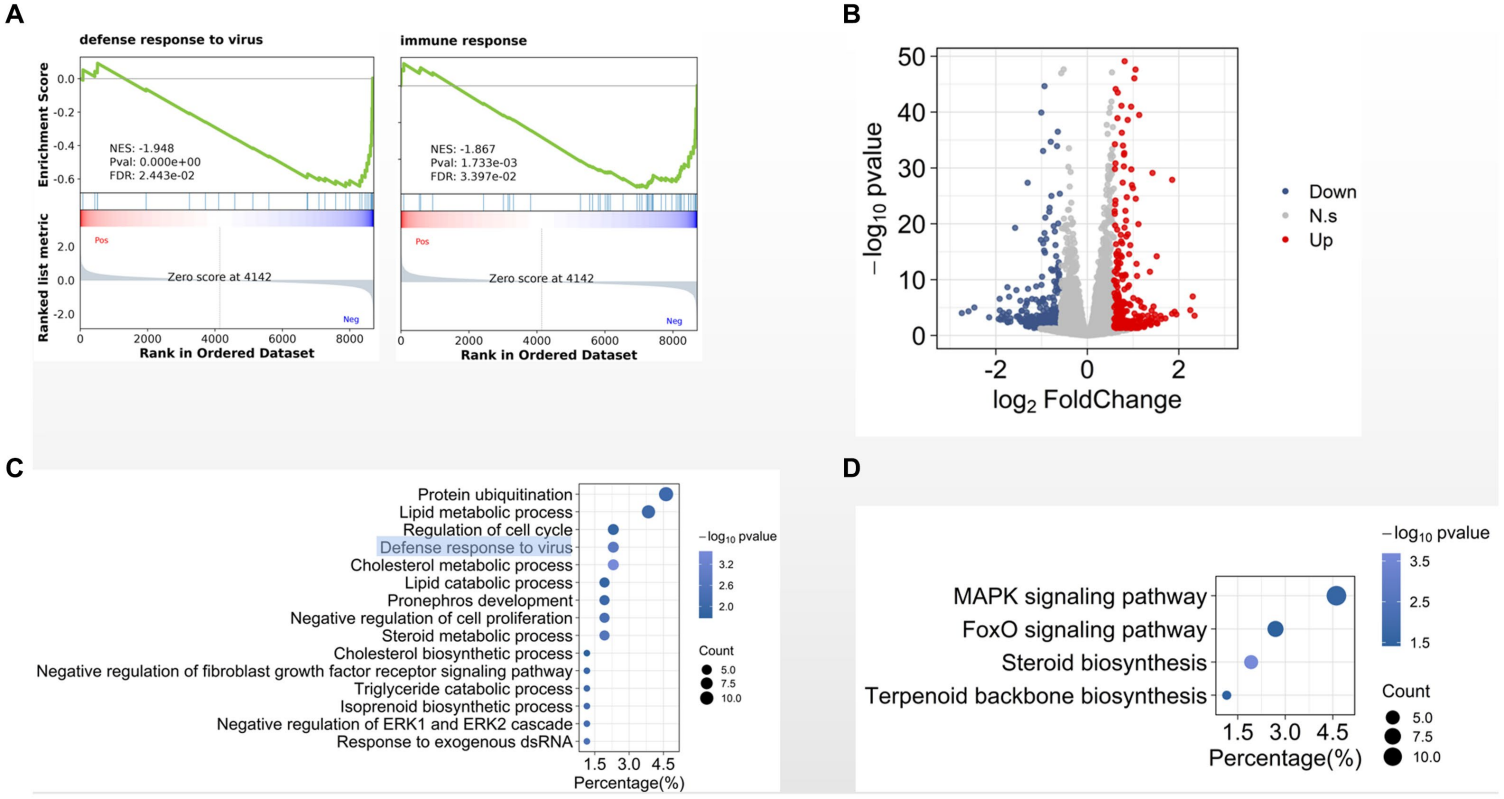
Transcriptional programs in GCRV-infected CIK. **(A)** Enrichment plot for defense- and immune-related genes. **(B)** Volcano plot of statistically significant differentially expressed genes in early infection. **(C, D)**. GO **(C)** and KEGG pathway **(D)** analyses of differentially expressed genes.

Totally we identified 15,255 expressed genes in three groups. We then used gene set enrichment analysis (GSEA) to compare the transcriptome data between CIK cells 8 hpi and the MOCK-infected cells. We found that several gene sets were significantly enriched in cells 8 hpi comparing with the MOCK, such as defense response to virus, immune response, and cholesterol metabolic process (Figure 2A). Comparing with MOCK, we identified 675 differentially expressed genes in CIK cells 8 hpi (Figure 2B). Gene ontology (GO) analysis indicates that the biological processes of defense response to virus, cholesterol metabolic process (Figure 2C) and mitogen-activated protein kinase (MAPK) signaling pathway (Figure 2D) are significantly enriched in these differentially expressed genes.

### DNA methylation pattern in grass carp reovirus early infected *Ctenopharyngodon idellus* kidney cells

**Figure 3:**
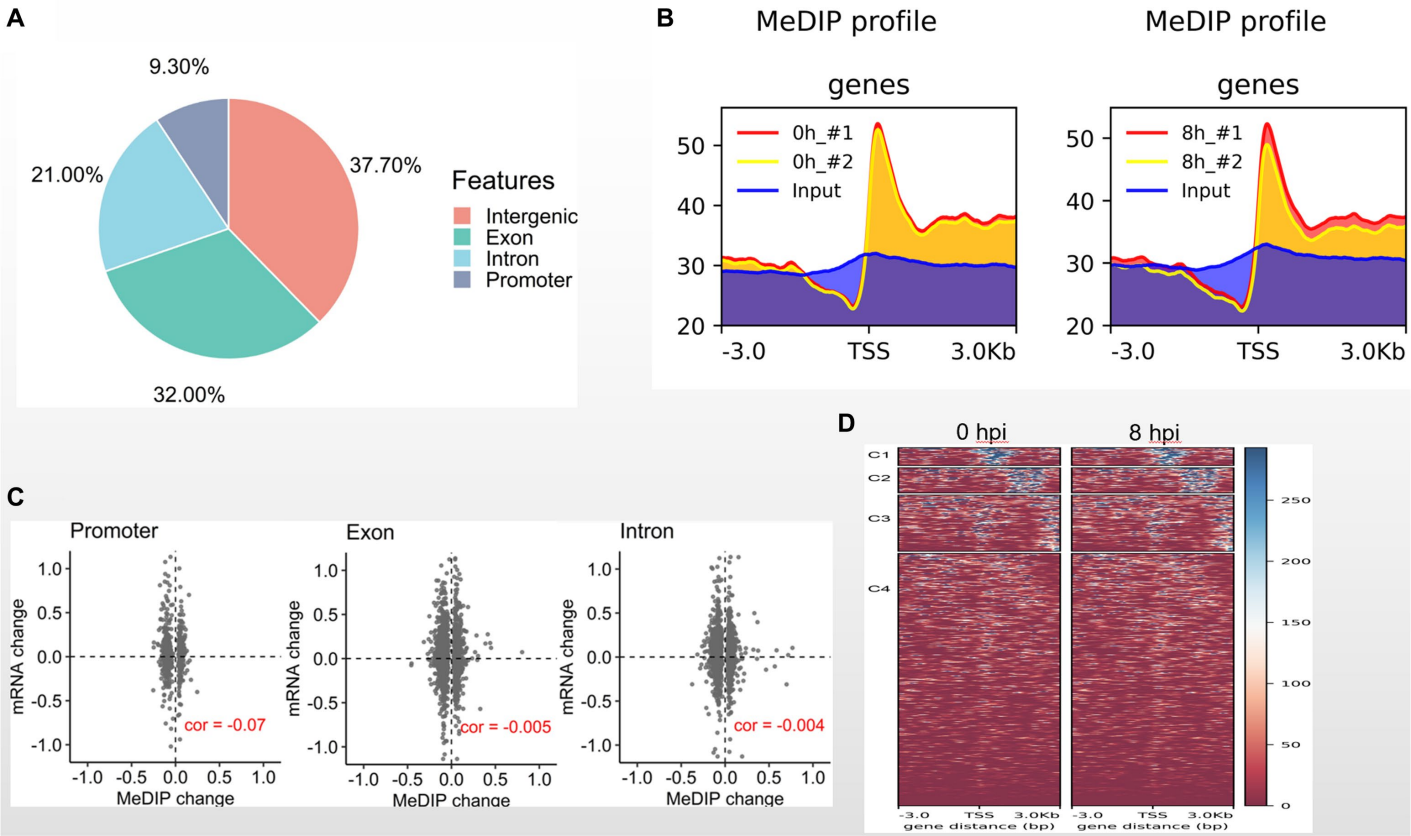
DNA methylome in early GCRV infection. **(A)** Genomic distribution of methylated regions in CIK cells. **(B)** DNA methylated signal around TSS regions. **(C)** Correlation between transcriptional signal and methylated regions. **(D)** DNA methylation heatmaps of MOCK and two biological replicates of 8 hpi group.

It has been reported that DNA methylation contributes to resistance to GCRV infection (Shang et al., 2017). Here, we asked whether DNA methylation is involved in GCRV early infection. To address this issue, we performed MeDIP-seq analysis with MOCK and CIK cells 8 hpi. We totally generated 232 million sequence reads, and 96% are mappable. The data sets of biological replicates are highly correlated (Supplementary Figure). Among 9,426 identified methylation sites 37.7% are located in intergenic regions, 32% in exons, 21% in introns and only 9.3% in promoter regions (Figure 3A). We examined DNA methylation signal around transcription starting site (TSS) and found the obviously enriched methylation signal at TSS regions both in MOCK and 8 hpi groups (Figure 3B). It is well recognized that DNA methylation is negatively associated with gene expression. We then examined the correlation between methylated genomic regions and transcription levels. We observed that the methylated regions at promoters, exons and introns are weakly and negatively correlated with transcription (Figure 3C). Compared with the MOCK, we found the DNA methylation of CIK cells 8 hpi is very similar to MOCK (Figure 3D), suggesting that DNA methylation pattern is less functionally involved in early GCRV infection.

### Alternative polyadenylation profile in grass carp reovirus early infected *Ctenopharyngodon idellus* kidney cells

**Figure 4:**
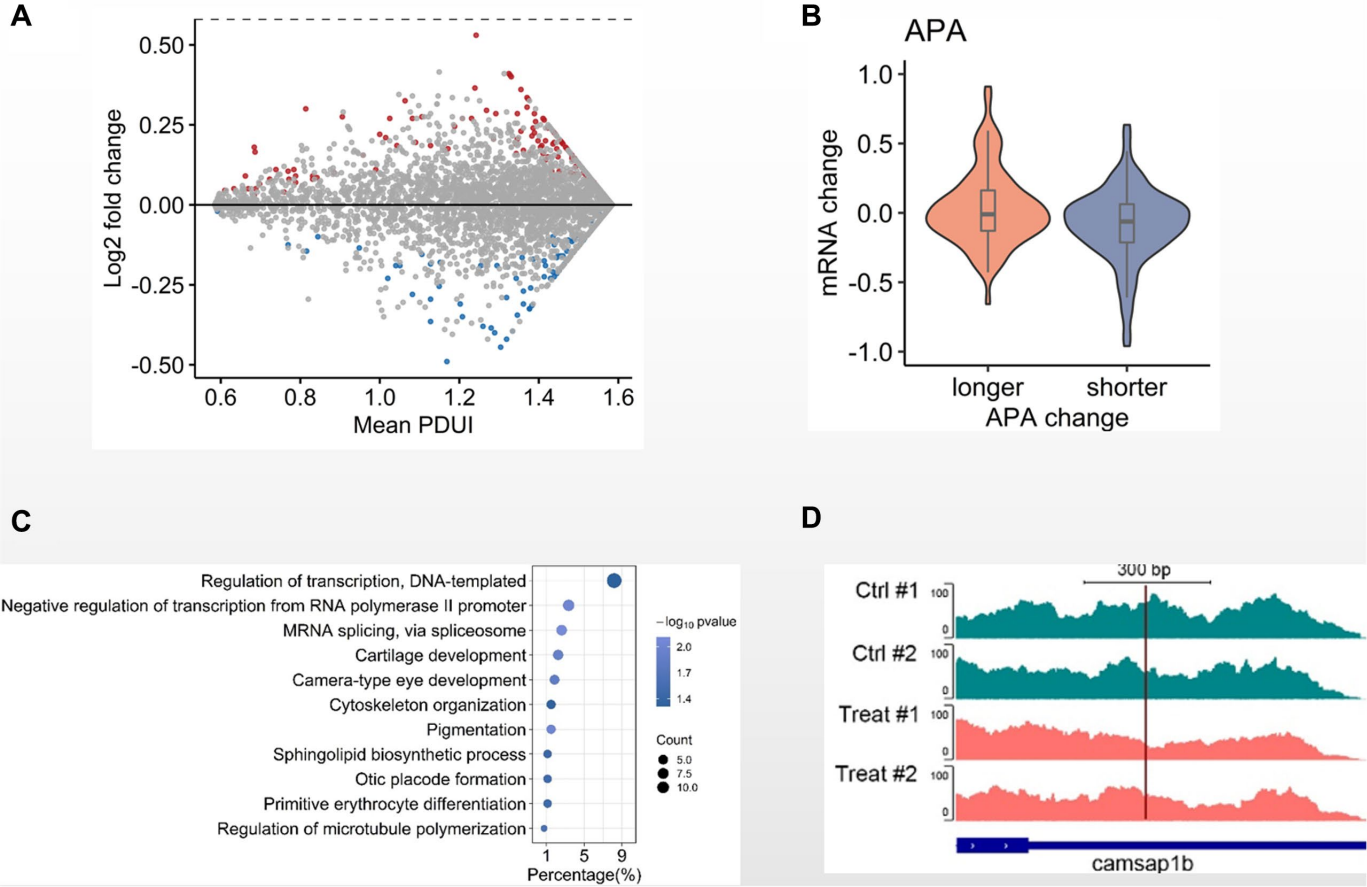
Alternative polyadenylation analysis in GCRV-infected CIK cells. **(A)** MA-plot depicted 3’UTRs of transcripts demarcated by DaPars-defined APA sites. The 3’UTRs were significantly shortened (blue) or lengthened (red) in CIK cells 8 hpi when compared the MOCK (value of *p* < 0.05). **(B)** Violin plot showing transcription level change of genes with altered 3′ UTR in GCRV-infected CIK cells vs. MOCK comparison. **(C)** GO items of biological processes enriched in genes with altered 3’UTR. **(D)** Genomic view of APA site usage preference at *Camsap1b* 3’UTR in IGV browser, showing the transcriptional density shifting to the proximal APA upon GCRV infection.

Since DNA methylation is less involved in GCRV early infection, we next investigated other mechanisms. Alternative polyadenylation (APA) modulates gene expression and has been reported to be involved in antiviral response. We then examined the APA patterns between MOCK and 8 hpi group. Comparing with the MOCK, we identified 404 genes with the APA-derived altered 3’UTRs, including 201 genes with lengthened 3’UTRs and 203 genes with shortened 3’UTRs (Figure 4A). When examining the 3’UTR alterations and transcription levels, we observed that the overall transcription levels of the genes with shortened 3’UTRs is higher than the those with lengthened 3’UTRs (Figure 4B). Through GO analysis with the genes containing altered 3’UTRs we found the biological processes of cytoskeleton organization and regulation of microtubule polymerization are statistically enriched (Figure 4C). In particular, we observed that *Camsap1b*, a gene involved in microtubule formation and stability, preferentially utilized the proximal poly(A) sites in GRCV-infected CIK cells when comparing MOCK (Figure 4D). These observations suggest that alternative poly(A) usage is potentially involved in the early infection of CIK cells.

## Discussion

Viral infections involve intensive interactions between viruses and host cells. As obligate intracellular parasites, viruses misappropriate host cellular machinery to allow their replication; while host cells also orchestrate the transcriptional programs to repress viral infection. For example, in our previous studies we found that aquareovirus NS38 (the GCRV nonstructural protein expressed in host cells as early as 3 h post infection) interacts with host translation initiation factor eIF3A for virus replication (Shao et al., 2013; Zhang et al., 2019). Meanwhile, host innate immune response would be activated after virus infection. Consist with the reported studies (Chen et al., 2012; Shi et al., 2014; Wan and Su, 2015; Dang et al., 2016; Xu et al., 2016; Chen et al., 2018), we observed that the host genes related to defense response to virus and immune response are differentially expressed in CIK cells 8 hpi (Figures 2A,C). In addition to these immune-related genes, we found the genes involved in cholesterol metabolic process and cholesterol biosynthetic process are also activated (Figure 2C), supporting our previous report that cellular membrane cholesterol is required for GCRV productive infection (Zhang et al., 2018). Activation of MAPK signaling pathway has been reported to be required for cell entry of avian reovirus (Huang et al., 2011). Interestingly, in this study we found that this pathway is most significantly enriched among all identified cellular signaling pathways (Figure 2D), suggesting MAPK signaling pathway is involved in GCRV infection.

DNA methylation has been reported to control the resistance and susceptibility to GCRV infection in CIK cells (Shang et al., 2017). In this study we examined the DNA methylome of CIK cells 8 hpi and found that the DNA methylation pattern of infected cells is very similar to the MOCK (Figure 3D). The statistically enriched biological processes of differentially methylated genes do not include defense response to virus or immune response (data not shown). These findings indicate that DNA methylation is less functionally involved in early GCRV infection.

Alternative polyadenylation functionally contributes to antiviral immune response (Jia et al., 2017). Some poly(A) binding proteins are the components of stress granules, the membrane-less ribonucleoprotein (RNP)-based cellular compartments in the cytoplasm triggering antiviral immune response. Moreover, alternative polyadenylation is involved in chronically infected disease (Su et al., 2001). Previously, we have performed extensive alternative polyadenylation analysis to understand its functional relevance in tumorigenesis (Lai et al., 2015; Tan et al., 2018, 2021). In this study we identified a considerable number of genes that selectively utilize alternative poly(A) sites in GCRV-infected CIK cells (Figures 4A,B). Among the genes with altered 3’UTRs we identified the biological processes of cytoskeleton organization, regulation of microtubule polymerization (Figures 4C,D). Interestingly, our recent study reported that microtubules are required for productive GCRV infection (Zhang et al., 2020), which is similar to MRV infection (Mainou et al., 2013).These observations suggest that alternative polyadenylation is potentially involved in GCRV early infection. Taken together, our study provides evidence of molecular events during early infection of dsRNA viruses for understanding their pathogenesis.

## Data availability statement

The datasets presented in this study can be found in online repositories. The names of the repository/repositories and accession number(s) can be found in the article/Supplementary material.

## Ethics statement

The animal studies were approved by the Research Ethics Committee of the Shanghai Jiao Tong University. The studies were conducted in accordance with the local legislation and institutional requirements. Written informed consent was obtained from the owners for the participation of their animals in this study.

## Author contributions

ST: Conceptualization, Data curation, Writing – review & editing. JZ: Data curation, Resources, Writing – review & editing. YP: Data curation, Software, Writing – review & editing, Formal analysis, Investigation. WD: Investigation, Writing – review & editing. JY: Writing – review & editing, Formal analysis. QF: Conceptualization, Writing – original draft, Writing – review & editing, Funding acquisition.

## Footnotes

^1^ http://www.ebi.ac.uk/arrayexpress/

^2^ https://www.ebi.ac.uk/biostudies/arrayexpress/studies/E-MTAB-13002

^4^ https://www.ebi.ac.uk/biostudies/arrayexpress/studies/E-MTAB-13003

^6^ https://github.com/broadinstitute/picard

^7^ https://david.ncifcrf.gov/home.jsp

## References

[ref1] Anders S. Huber W. (2010). Differential expression analysis for sequence count data. Genome Biol. 11:R106. doi: 10.1186/gb-2010-11-10-r106, PMID: 20979621 PMC3218662

[ref2] Berger S. L. Kouzarides T. Shiekhattar R. Shilatifard A. (2009). An operational definition of epigenetics. Genes Dev. 23, 781–783. doi: 10.1101/gad.1787609, PMID: 19339683 PMC3959995

[ref3] Chen G. He L. Luo L. Huang R. Liao L. Li Y.. (2018). Transcriptomics sequencing provides insights into understanding the mechanism of grass carp Reovirus infection. Int. J. Mol. Sci. 19:488. doi: 10.3390/ijms19020488 29415502 PMC5855710

[ref4] Chen J. Li C. Huang R. Du F. Liao L. Zhu Z.. (2012). Transcriptome analysis of head kidney in grass carp and discovery of immune-related genes. BMC Vet. Res. 8:108. doi: 10.1186/1746-6148-8-108, PMID: 22776770 PMC3505460

[ref5] Dang Y. Xu X. Shen Y. Hu M. Zhang M. Li L.. (2016). Transcriptome analysis of the innate immunity-related complement system in spleen tissue of *Ctenopharyngodon idella* infected with *Aeromonas hydrophila*. PLoS One 11:e0157413. doi: 10.1371/journal.pone.0157413, PMID: 27383749 PMC4934786

[ref6] Fang Q. Ke L. H. Cai Y. Q. (1989). Growth characteristics and high titer culture of grass carp hemorrhage virus (GCHV)-873 in vitro. Virol. Sin. 3, 315–319.

[ref7] Guo H. Sun X. Yan L. Shao L. Fang Q. (2013). The NS16 protein of aquareovirus-C is a fusion-associated small transmembrane (FAST) protein, and its activity can be enhanced by the nonstructural protein NS26. Virus Res. 171, 129–137. doi: 10.1016/j.virusres.2012.11.011, PMID: 23201583

[ref8] Guo H. Zhang J. Wang Y. Bu C. Zhou Y. Fang Q. (2017). Comparative proteomic analysis of lysine acetylation in fish CIK cells infected with Aquareovirus. Int. J. Mol. Sci. 18:2419. doi: 10.3390/ijms18112419, PMID: 29135940 PMC5713387

[ref9] Hino R. Uozaki H. Murakami N. Ushiku T. Shinozaki A. Ishikawa S.. (2009). Activation of DNA methyltransferase 1 by EBV latent membrane protein 2A leads to promoter hypermethylation of PTEN gene in gastric carcinoma. Cancer Res. 69, 2766–2774. doi: 10.1158/0008-5472.CAN-08-3070, PMID: 19339266

[ref10] Huang W. R. Wang Y. C. Chi P. I. Wang L. Wang C. Y. Lin C. H.. (2011). Cell entry of avian reovirus follows a caveolin-1-mediated and dynamin-2-dependent endocytic pathway that requires activation of p38 mitogen-activated protein kinase (MAPK) and Src signaling pathways as well as microtubules and small GTPase Rab5 protein. J. Biol. Chem. 286, 30780–30794. doi: 10.1074/jbc.M111.257154, PMID: 21705803 PMC3162439

[ref11] Jia X. Yuan S. Wang Y. Fu Y. Ge Y. Ge Y.. (2017). The role of alternative polyadenylation in the antiviral innate immune response. Nat. Commun. 8:14605. doi: 10.1038/ncomms14605, PMID: 28233779 PMC5333124

[ref12] Ke L. H. Fang Q. Cai Y. Q. (1990). Characteristics of a novel isolate of grass carp hemorrhagic virus. Acta Hydrobiologica Sinica 14, 153–159.

[ref13] Kechin A. Boyarskikh U. Kel A. Filipenko M. (2017). cutPrimers: a new tool for accurate cutting of primers from reads of targeted next generation sequencing. J. Comput. Biol. 24, 1138–1143. doi: 10.1089/cmb.2017.0096, PMID: 28715235

[ref14] Kim D. Paggi J. M. Park C. Bennett C. Salzberg S. L. (2019). Graph-based genome alignment and genotyping with HISAT2 and HISAT-genotype. Nat. Biotechnol. 37, 907–915. doi: 10.1038/s41587-019-0201-4, PMID: 31375807 PMC7605509

[ref15] Lai D. P. Tan S. Kang Y. N. Wu J. Ooi H. S. Chen J.. (2015). Genome-wide profiling of polyadenylation sites reveals a link between selective polyadenylation and cancer metastasis. Hum. Mol. Genet. 24, 3410–3417. doi: 10.1093/hmg/ddv089, PMID: 25759468

[ref16] Langmead B. Salzberg S. L. (2012). Fast gapped-read alignment with bowtie 2. Nat. Methods 9, 357–359. doi: 10.1038/nmeth.1923, PMID: 22388286 PMC3322381

[ref17] Liao Y. Smyth G. K. Shi W. (2014). featureCounts: an efficient general purpose program for assigning sequence reads to genomic features. Bioinformatics 30, 923–930. doi: 10.1093/bioinformatics/btt656, PMID: 24227677

[ref18] Lupianni B. Subramanian K. Samal S. K. (1995). Aquareoviruses. Annu. Rev. Fish Dis. 5, 175–208. doi: 10.1016/0959-8030(95)00006-2

[ref19] Mainou B. A. Zamora P. F. Ashbrook A. W. Dorset D. C. Kim K. S. Dermody T. S. (2013). Reovirus cell entry requires functional microtubules. MBio 4:13. doi: 10.1128/mBio.00405-13 PMC3705452 23820395

[ref20] Masamha C. P. Xia Z. Yang J. Albrecht T. R. Li M. Shyu A. B.. (2014). CFIm25 links alternative polyadenylation to glioblastoma tumour suppression. Nature 510, 412–416. doi: 10.1038/nature13261, PMID: 24814343 PMC4128630

[ref21] Pion M. Jaramillo-Ruiz D. Martínez A. Muñoz-Fernández M. A. Correa-Rocha R. (2013). HIV infection of human regulatory T cells downregulates Foxp3 expression by increasing DNMT3b levels and DNA methylation in the FOXP3 gene. AIDS 27, 2019–2029. doi: 10.1097/QAD.0b013e32836253fd, PMID: 24201117

[ref22] Rangel A. A. C. Rockemann D. D. Hetrick F. M. Samal S. K. (1999). Identification of grass carp haemorrhage virus as a new genogroup of aquareovirus. J. Gen. Virol. 80, 2399–2402. doi: 10.1099/0022-1317-80-9-2399, PMID: 10501493

[ref23] Robinson M. D. McCarthy D. J. Smyth G. K. (2010). edgeR: a Bioconductor package for differential expression analysis of digital gene expression data. Bioinformatics 26, 139–140. doi: 10.1093/bioinformatics/btp616, PMID: 19910308 PMC2796818

[ref24] Shang X. Yang C. Wan Q. Rao Y. Su J. (2017). The destiny of the resistance/susceptibility against GCRV is controlled by epigenetic mechanisms in CIK cells. Sci. Rep. 7:4551. doi: 10.1038/s41598-017-03990-5, PMID: 28674382 PMC5495752

[ref25] Shao L. Guo H. Yan L. M. Liu H. Fang Q. (2013). Aquareovirus NS80 recruits viral proteins to its inclusions, and its C-terminal domain is the primary driving force for viral inclusion formation. PLoS One 8:e55334. doi: 10.1371/journal.pone.0055334, PMID: 23424630 PMC3570539

[ref26] Shi M. Huang R. Du F. Pei Y. Liao L. Zhu Z.. (2014). RNA-seq profiles from grass carp tissues after reovirus (GCRV) infection based on singular and modular enrichment analyses. Mol. Immunol. 61, 44–53. doi: 10.1016/j.molimm.2014.05.004, PMID: 24865419

[ref27] Su Q. Wang S. F. Chang T. E. Breitkreutz R. Hennig H. Takegoshi K.. (2001). Circulating hepatitis B virus nucleic acids in chronic infection: representation of differently polyadenylated viral transcripts during progression to nonreplicative stages. Clin. Cancer Res. 7, 2005–2015. PMID: 11448918

[ref28] Taiwo O. Wilson G. A. Morris T. Seisenberger S. Reik W. Pearce D.. (2012). Methylome analysis using MeDIP-seq with low DNA concentrations. Nat. Protoc. 7, 617–636. doi: 10.1038/nprot.2012.012, PMID: 22402632

[ref29] Tan S. Li H. Zhang W. Shao Y. Liu Y. Guan H.. (2018). NUDT21 negatively regulates PSMB2 and CXXC5 by alternative polyadenylation and contributes to hepatocellular carcinoma suppression. Oncogene 37, 4887–4900. doi: 10.1038/s41388-018-0280-6, PMID: 29780166

[ref30] Tan S. Zhang M. Shi X. Ding K. Zhao Q. Guo Q.. (2021). CPSF6 links alternative polyadenylation to metabolism adaption in hepatocellular carcinoma progression. J. Exp. Clin. Cancer Res. 40:85. doi: 10.1186/s13046-021-01884-z, PMID: 33648552 PMC7923339

[ref31] Tsai C. L. Li H. P. Lu Y. J. Hsueh C. Liang Y. Chen C. L.. (2006). Activation of DNA methyltransferase 1 by EBV LMP1 involves c-Jun NH(2)-terminal kinase signaling. Cancer Res. 66, 11668–11676. doi: 10.1158/0008-5472.CAN-06-2194, PMID: 17178861

[ref32] Wan Q. Su J. (2015). Transcriptome analysis provides insights into the regulatory function of alternative splicing in antiviral immunity in grass carp (*Ctenopharyngodon idella*). Sci. Rep. 5:12946. doi: 10.1038/srep12946, PMID: 26248502 PMC4528194

[ref33] Wang Y. Lu Y. Zhang Y. Ning Z. Li Y. Zhao Q.. (2015). The draft genome of the grass carp (Ctenopharyngodon idellus) provides insights into its evolution and vegetarian adaptation. Nat. Genet. 47, 625–631. doi: 10.1038/ng.3280, PMID: 25938946

[ref34] Xu B. H. Zhong L. Liu Q. L. Xiao T. Y. Su J. M. Chen K. J.. (2016). Characterization of grass carp spleen transcriptome during GCRV infection. Genet. Mol. Res. 15:gmr6650. doi: 10.4238/gmr.15026650, PMID: 27173223

[ref35] Yan S. Zhang J. Guo H. Yan L. Chen Q. Zhang F.. (2015). VP5 autocleavage is required for efficient infection by in vitro-recoated aquareovirus particles. J. Gen. Virol. 96, 1795–1800. doi: 10.1099/vir.0.000116, PMID: 25742690

[ref36] Youngblood B. Noto A. Porichis F. Akondy R. S. Ndhlovu Z. M. Austin J. W.. (2013). Cutting edge: prolonged exposure to HIV reinforces a poised epigenetic program for PD-1 expression in virus-specific CD8 T cells. J. Immunol. 191, 540–544. doi: 10.4049/jimmunol.1203161, PMID: 23772031 PMC3702641

[ref37] Zhang F. Guo H. Chen Q. Ruan Z. Fang Q. (2020). Endosomes and Microtubles are required for productive infection in Aquareovirus. Virol. Sin. 35, 200–211. doi: 10.1007/s12250-019-00178-1, PMID: 31858455 PMC7198692

[ref38] Zhang J. Guo H. Zhang F. Chen Q. Chang M. Fang Q. (2019). NS38 is required for aquareovirus replication via interaction with viral core proteins and host eIF3A. Virology 529, 216–225. doi: 10.1016/j.virol.2019.01.029, PMID: 30735905

[ref39] Zhang F. Guo H. Zhang J. Chen Q. Fang Q. (2018). Identification of the caveolae/raft-mediated endocytosis as the primary entry pathway for aquareovirus. Virology 513, 195–207. doi: 10.1016/j.virol.2017.09.019, PMID: 29102889

[ref40] Zhang Y. Liu T. Meyer C. A. Eeckhoute J. Johnson D. S. Bernstein B. E.. (2008). Model-based analysis of ChIP-Seq (MACS). Genome Biol. 9:R137. doi: 10.1186/gb-2008-9-9-r137, PMID: 18798982 PMC2592715

