# Supplementary material for: Integrative transcriptome analysis reveals alternative polyadenylation potentially contributes to GCRV early infection
Source: Front Microbiol. 2023 Nov 3;14:1269164. doi: 10.3389/fmicb.2023.1269164 (PMC10656684; doi:10.3389/fmicb.2023.1269164)
Supplement: Supplementary file 1 [file Image_1.pdf]

Supplementary information

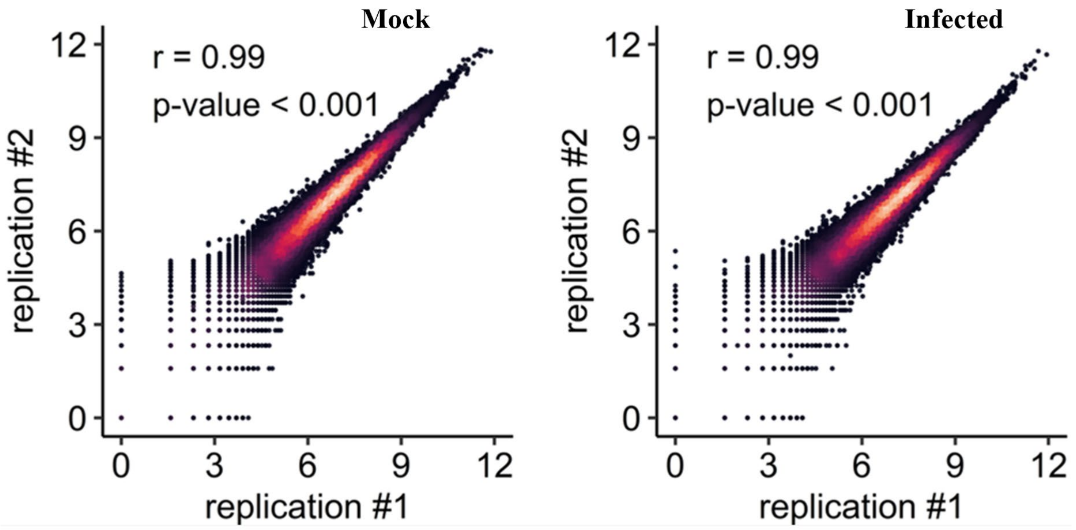

Supplementary Figure. Reproducibility characterization of MeDIP-seq data sets by scatterploting binned MeDIP-Seq signals in form of tag counts in 1-kb bins correlation was computed using whole genome data.
